# Resistance characteristics of CTX-M type *Shigella flexneri* in China

**DOI:** 10.1042/BSR20191741

**Published:** 2019-09-24

**Authors:** Fengzhi Bian, Mingxiao Yao, Hongmei Fu, Guangying Yuan, Shuzhi Wu, Yuguo Sun

**Affiliations:** 1 Department of Clinical Laboratory, The Fourth People’s Hospital of Jinan, Jinan, Shandong Province 250031, China; 2 Department of Viral Infectious Diseases Control and Prevention, Shandong Center for Disease Control and Prevention, Jinan, Shandong Province 250014, China; 3 Jinan Blood Supply and Security Center of Shandong Province, Jinan, Shandong Province 250023, China; 4 Department of Clinical Laboratory, Jinan Infectious Disease Hospital Affiliated to Shandong University, Jinan, Shandong Province 250021, China; 5 Collaborative Innovation Center for the Origin and Control of Emerging Infectious Diseases, Taishan Medical University, Taian, Shandong Province 271016, China

**Keywords:** antimicrobial resistance, ESBLs, genotype, Shigella flexneri

## Abstract

The present study was to identify the drug resistance, resistance mechanism and the extended-spectrum β-lactamase (ESBLs) genotypes of *Shigella flexneri* (*S. flexneri*) in Jinan. Susceptibility tests were performed by MIC-determination. The genotypes of β-lactamase were identified using PCR and DNA sequencing. The resistance transfer ability of the ESBL-producing strains was examined by conjugation tests. A total of 105 *S. flexneri* isolates were collected, and 34 (32.4%) were ESBL-producing isolates. All ESBL-producing isolates were susceptible to cefoxitin and imipenem, and 35.3% isolates were resistant to ciprofloxacin. ESBL-producing isolates showed high level resistant to ampicillin (100%), cefotaxime (100%), tetracycline (100%), chloramphenicol (100%), trimethoprim/sulfamethoxazole (100%), ceftazidime (73.5%) and cefepime (73.5%). Three types of β-lactamase genes (*bla*TEM, *bla*OXA and *bla*CTX-M) were identified in all ESBL-producing isolates, and the genotypes were confirmed as *bla*TEM-1 (23/34), *bla*OXA-30 (34/34), *bla*CTX-M-14 (9/34) and *bla*CTX-M-15 (25/34) by sequencing. In conclusion, the *Shigella* strains isolated in Jinan are cross-resistant and multi-drug resistant. The main genotypes of ESBLs are CTX-M-14 and CTX-M-15.

## Introduction

*Shigella* is a highly infectious intestinal bacteria that can cause serious harm. According to DuPont et al. [1], ingestion of 10–100 *Shigella* dysentery can make healthy individuals ill. *Shigella’s* endotoxin, exotoxin, enterotoxin and temperature regulation genes can cause fever, bloody purulent stool, abdominal cramps in infected individuals. Some patients can have very severe symptoms including hemolytic uremic syndrome, hypoglycemia, hyponatremia, intestinal perforation, seizures, encephalopathy and even death while others experience self-limiting illness. According to WHO in 1999, there are 165 million dysentery patients around the world every year, with about 162 million in the developing world. Dysentery was the cause of death for 1.1 million people, mainly children under the age of 5 [2]. *Shigella* was named by WHO in 1996 as a life threatening bacteria due to its growing resistance to treatment [3] and the morbidity associated with economic conditions, public health, living habits and epidemic *Shigella* serotypes. Bacillary dysentery is a serious public health problem in China [4], considered the third most dangerous among infectious diseases. Bacterial dysentery must be controlled through eliminating the source of infection, cutting off the route of transmission and protecting the vulnerable groups in time. However, in recent years, *Shigella* has become more drug resistant, causing inefficient treatment. Those *Shigella* patients who protracted course of disease are a primary source of infection in others, causing great difficulties for disease control and prevention and clinical treatment.

Bacterial drug resistance seriously impedes the efficacy of clinical treatment, increasing the cost of treatment, shortening the application period for new drugs and increasing the cost of drug development. In the 1950s, sulfonamides were effective for treatment of shigellosis, but soon produced drug resistance. Research indicated that the gene cassettes dfrA1, sat1 and aadA1 carried by *Shigella* encode resistance to sulfonamides, streptomycin and aminoglycoside antibiotics [5,6]. *Shigella* has been resistant to tetracycline, chloramphenicol and ampicillin since the 1970s; studies [7] show that in the R plasmid-mediated resistance of *Shigella* to these antibiotics, the transmissible plasmid carried the corresponding resistance genes. S*higella* developed resistance to quinolones at the beginning of the 1980s, which has been attributed to its gyrase A subunit (gyra) mutation and topoisomerase gene (parc) mutation. The third-generation cephalosporins have been highly effective against *Shigella* in the clinic up until 1999, when Ahamed & Kundul [8] first observed in India that *Shigella* developed SHV-11 type extended-spectrum β-lactamase (ESBLs), leading to cross resistance to penicillin and first-generation, second-generation and third-generation cephalosporins and monoamide antibiotics. Researchers in France then found SHV-2 type ESBLs, those in Japan found TOHO-1 type ESBLs, and those studying populations in Argentina found PER-2 type ESBLs. South Korea, Turkey, Lebanon and other countries reported CTX-M type ESBLs, which the main genotypes were being CTX-M-2, CTX-M-3, CTX-M-14 and CTX-M-15. The main genotypes in China were CTX-M-3, CTX-M-14 and CTX-M-15 [8–10]. The current paper presents results of research conducted in China between 2011 and 2016, analyzing the resistance, popular genotypes and resistant gene dissemination of clinical sporadic ESBLs-producing *Shigella* and the difference between CTX-M-14 and CTX-M-15 in affecting the capability of antibiotics.

## Materials and methods

### Bacterial isolates

All isolates were obtained from clinic patients with diarrhea in China from 2011 to 2016 and identified by ID 32E and serotyping as *Shigella flexneri (S. flexneri)*. Quality control strains of *Escherichia coli* ATCC 25922, *Klebsiella pneumoniae* ATCC 700603 and *E. coli* K12RifRLac- were presented by Doctor Xu Yuanhong of the First Affiliated Hospital of Anhui Medical University. The research has been carried out in accordance with the World Medical Association Declaration of Helsinki*, and that all subjects provided written informed consent.

### ESBLs screening and confirmation

ESBLs screening and confirmation were done to the standard of CLSI recommendations for *K. pneumoniae, Klebsiella oxytoca* and *E. coli*. Highly suspicious strains were identified by the bacteriostatic ring (≤27 mm to cefotaxime and ≤22 mm to ceftazidime) and confirmed by the double disk synergy test. In those drugs with clavulanic acid, as opposed to those without clavulanic acid, an inhibition zone diameter of ≥5 mm may be a preliminary indication of ESBL-producing strains.

### Antimicrobial susceptibility testing

The Kirby–Bauer (K–B) disk diffusion method and MIC determination were utilized as the method and criterion for antimicrobial susceptibility testing, respectively, according to the CLSI 2010 version of M100-S20 regulations.

### Extraction of DNA

Genomic DNA was extracted from the isolates using TIANamp Bacteria DNA kit (Tiangen Biotech (Beijing) Co., Ltd.), All operations are carried out in accordance with the Kit specification.

### Primer synthesis

According to the primer sequence described in Table 1, TEM, SHV, OXA, the CTX-M-1 group, the CTX-M-2 group, the CTX-M-8 group, the CTX-M-9 group and the CTX-M-25 group-encoding gene primers were synthesized.

**Table 1:** Sequence and annealing temperature of β-lactamase gene primers

Primer	Sequence (5′→3′)	Product size	Annealing temp (°C)	Reference
TEM	FP: CCCTGGTAAATGCTTC	919	45	[1]
	RP: GAGTAAACTTGGTCTG			
SHV	FP: GGTTATGCGTTATATTCGCC	864	58	[3]
	RP: TTAGCGTTGCCAGTGCTC			
OXA	FP: ACACAATACATATCAACTTCGC	885	50	[3]
	RP: AGTGTGTTTAGAATGGTGATC			
CTX-M-1 group	FP: CGT CAC GCT GTT GTT AGG AA	780	50	[4]
	RP: ACG GCT TTC TGC CTT AGG TT			
CTX-M-2 group	FP: TTA ATG ATG ACT CAG AGC ATT C	902	49	[5]
	RP: GAT ACC TCG CTC CAT TTA TTT			
CTX-M-8 group	FP: ACT TCA GCC ACA CGG ATT CA	948	50	[5]
	RP: AAG TGG AGC GAC AGA GC			
CTX-M-9 group	FP: CGG AAGCAGTCTAAATTC TTCGTGAAATAG	1160	54	[1]
	RP: CGG GCC AGT TGG TGA TTT GA			
CTX-M-25 group	FP: GTA AGG CGG GCG ATG TTA AT	856	50	[5]
	RP: AAC CGT CGG TGA CAA TTC TG			

### PCR amplification and DNA sequencing

The PCR reaction system was a mixture of 0.15 μl of Taq DNA polymerase (5 U/μl), 3 μl of 10× PCR buffer (Mg^2+^) and 2.4 μl of dNTP mixture (2.5 mM). Next, 1.5 μl of forward and reverse primers (10 μM), 3 μl of DNA template and sterile double-distilled water were added to give a total reaction volume of 30 μl. The PCR amplification conditions included pre-denaturation at 94°C for 5 min; 35 cycles of 94°C for 30 s, annealing (at respective annealing temp) for 30 s and 72°C for 40 s; and final extension at 72°C for 5 min. The PCR products were analyzed by 1% agarose gel electrophoresis, and the positive PCR products were sent to be sequenced.

### Conjugation analysis

The ESBL-producing strains were specified as the donor bacteria, and *E. coli* K12RifRLac- was specified as the recipient bacteria. An individual colony of donor and recipient bacteria were inoculated in 1 ml of common broth. After incubation at 35°C for 6 h, 100 μl of donor bacteria and 100 μl of recipient bacteria were placed into a 500 μl sterile ordinary broth mix, conjugated for 2 h at 35°C, then the positive colony was picked up on a China blue plate containing cefotaxime (1 mg/l). The red colony could be used for further pure culture and identified by ID 32E. The genotype and MIC of the conjugation were determined as previously described.

### Relative hydrolysis rate determination

The CTX-M-14 and CTX-M-15 *Shigella* genotypes were in nutrient broth overnight then centrifuged at 4000 r/min for 15 min. The precipitate was washed three times with physiological brine and finally suspended in 2 ml of normal saline. The precipitate in saline was frozen and thawed eight times then centrifuged at 8000 r/min for 30 min. The supernatant was a ESBLs crude extract. Thirty microliters of the extract was then added to 3 ml of cephalosporin, diluting it to a final concentration of 0.1 mmol/l, and the OD value was determined by ultraviolet spectrophotometry. After a 15 min water bath at 37°C, the OD value was determined again. The enzymatic hydrolysis of 100% cefazolin was used as a benchmark to calculate the relative hydrolysis rate of each kind of cephalosporin (%) (the optimum wavelength of each would be: cefazolin, 265 nm; cefuroxime, 265 nm; cefotaxime, 257 nm; ceftazidime, 257 nm; aztreonam, 277 nm; cefepime, 261 nm; and cefoxitin, 260 nm). The relative hydrolysis rate = (ΔOD of other cephalosporins/ΔOD of cefazolin) × 100%.

## Results

### The isolated rate of ESBL-producing *Shigella*

In 105 strains of *S. flexneri*, 40 suspicious strains were screened by the K–B method, and a preliminary determination indicated that 34 strains were ESBL-producing. The genotyping method confirmed 34 strains (32.4%) were ESBL-producing.

### Antimicrobial susceptibility analysis

All 34 ESBL-producing *Shigella* strains were resistant to ampicillin, cefotaxime, tetracycline, chloramphenicol, sulfamethoxazole and trimethoprim; 28 strains were resistant to cefepime and ceftazidime, and 13 to ciprofloxacin. All 34 ESBL-producing *Shigella* strains were sensitive to cefoxitin, imipenem and gentamicin. The results are shown in Table 2.

**Table 2:** MIC of 34 ESBL-producing *S. flexneri* and corresponding conjugon (μg/ml)

Antimicrobial agents	Range of MIC	Crit. conc.	MIC of 34 ESBLs- producing *S. flexneri* (quantity)		MIC of 34 conjugon (quantity)	
			CTX-M-14type(9)	CTX-M-15type(25)	CTX-M-14type(9)	CTX-M-15type(25)
Ampicillin	0.016–256	16	>256(9)	>256(25)	>256(9)	>256(25)
Cefotaxime	0.016–256	2	32(7), >256(2)	>256(25)	16(2), 32(7)	>256(25)
Ceftazidime	0.016–256	8	1(7), 2(2)	16(2), 32(23)	0.5(1), 1(1), 2(7)	8(2), 16(23)
Cefoxitin	0.016–256	16	2(2), 4(7)	2(2), 4(23)	2(1), 4(8)	2(1), 4(24)
Cefepime	0.016–256	16	4(5), 8(4)	64(8), 128(9), 256(8)	1(3), 2(4), 4(2)	16(8), 64(14), 256(3)
Imipenem	0.06–32	8	0.25(7), 0.5(2)	0.25(23), 0.5(2)	0.125(7), 0.25(2)	<0.06(10), 0.125(15)
Ciprofloxacin	0.02–32	2	0.125(2), 4(7)	0.25(20), 4(5)	<0.02(9)	<0.02(25)
Gentamicin	0.016–128	8	1(5), 2(3), 32(1)	1(21), 2(4)	0.25(8), 0.5(1), 8(1)	0.25(15), 0.5(6), 1(4)
Chloramphenicol	0.016–256	16	128(3), >256(6)	64(11), 128(5), 256(9)	4(5), 8(4)	4(23), 8(2)
Tetracycline	0.016–256	8	128(1), 256(8)	128(2), 256(23)	1(8), 2(1)	1(23), 128(2)
Sulfamethoxazole	2–1024	64	>1024(9)	>1024(25)	4(2), 8(7)	4(7), 8(16), 16(2)
Trimethoprim	0.125–64	4	>64(9)	>64(25)	<0.125(9)	<0.125(25)

### Results of PCR amplification

**Figure 1:**
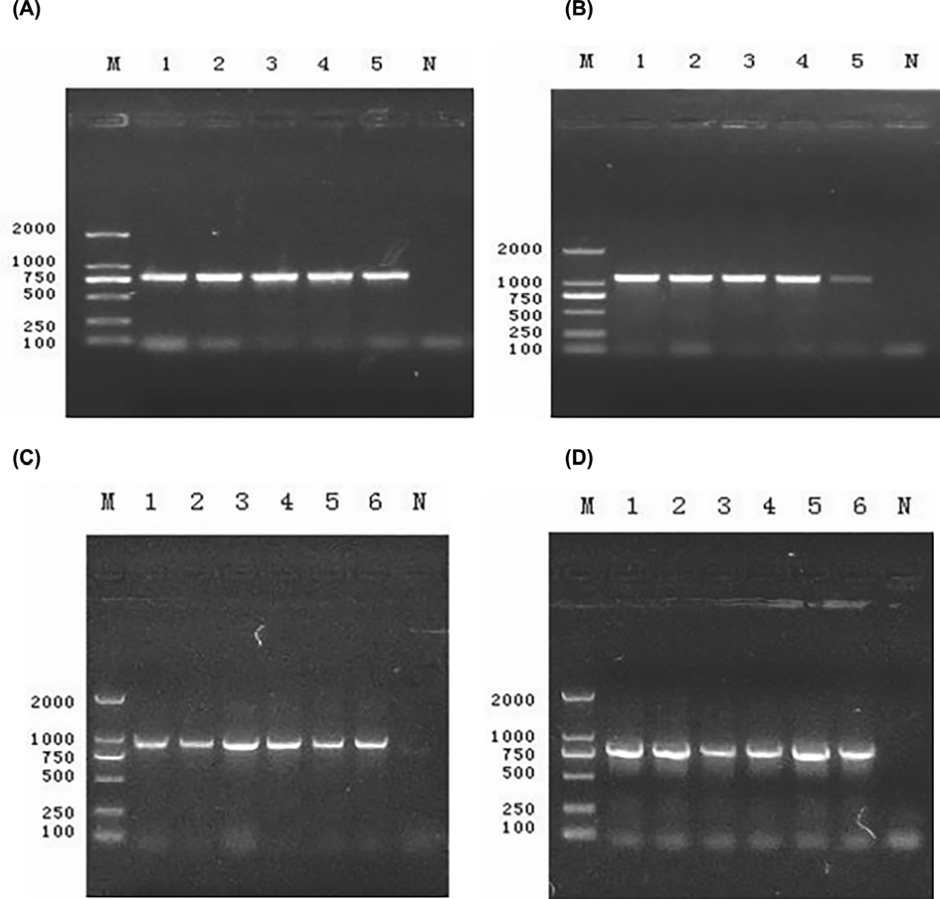
Electrophoretogram of *bla*CTX-M gene M: Marker D2000; 1, 2, 3, 4, 5 and 6: Random strain; N: *E. coli* ATCC 25922. (**A,B,C,D**): Electrophoretogram of CTX-M-1, CTX-M-9, TEM and OXA gene amplification product.

All 34 ESBL-producing *Shigella* strains were positive for the *bla*CTX-M gene, including 25 in the CTX-M-1 group (Figure 1A) and nine in the CTX-M-9 group (Figure 1B). Twenty-three strains were positive for *bla*TEM (Figure 1C) and 34 were positive for *bla*OXA (Figure 1D) but were not detected in SHV, CTX-M-2, CTX-M-8 or CTX-M-25 positive strains. Positive amplification products by DNA sequencing confirmed that the CTX-M-1 group was the *bla*CTX-M-15 gene, and the CTX-M-9 group was the *bla*CTX-M-14 gene that encoded ESBLs. The *bla*TEM and *bla*OXA genes were *bla*TEM-1 and *bla*OXA-30, respectively, encoding β-lactamase instead of ESBLs.

### Conjugation analysis

All 34 ESBL-producing *Shigella* conjugative transfer tests were positive, and the biochemical identification results of conjugation were the same as the recipient bacterium *E. coli* K12RifRLac^−^. PCR amplification confirmed that all strains transferred blaCTX-M and that the genotype was consistent with the donor bacteria. MIC results and conjugons are presented in Table 3.

**Table 3:** Relative hydrolysis rate determination of CTX-M Type *S. flexneri* (%)

Antimicrobial agents	CTX-M-14	CTX-M-15
Cefazolin	100	100
Cefuroxime	85.8	88.0
Cefotaxime	70.7	65.9
Ceftazidime	2.6	7.7
Aztreonam	0	7.2
Cefepime	51.9	73.7
Cefoxitin	0	0

## Discussion

With the wide application of third-generation cephalosporins, the ESBL-producing *Shigella* detection rate increased gradually [11]. In the present study, 32.4% (34/105) of *S. flexneri* strains produced ESBLs, which was more than the separation rate of 10.3% in this area during 2003–2006 [12]. These statistics indicate that there is an increasing trend of ESBL-producing *Shigella* in the district. One hand, with third-generation cephalosporins being used in clinical experience widely against the increasing multi-drug resistance of *Shigella*, the selective pressure caused resistance to grow more gradually. On the other hand, in the ESBL-producing Enterobacteriaceae (such as *E. coli* and *K. pneumoniae*) family, plasmids transferred the ESBLs resistance genes through transformation, transduction and conjugation. Conjugation analysis also confirmed the presence of conjugative plasmid in ESBL-producing *Shigella*, which carried the ESBLs resistance genes that had completely transferred to the receptor bacteria; three strains were also carrying the gentamicin and tetracycline resistance gene. Nicolas [13] found that *S. flexneri* has SHV-2 ESBLs, located in an 80-Kb plasmid; and Kim et al. [14] found that the ESBL-encoding gene of *Shigella sonnei* was located in the conjugative plasmid, with individual strains of two or three types of ESBLs. All of these results reveal that conjugative plasmid plays a key role in the transmission of ESBLs and other resistance genes, leading to horizontal transfer of the drug resistance genes and communication between the homologous and heterologous bacteria.

The first ESBLs of the CTX-M type were isolated in 1989 from *E. coli* [15]. The name CTX-M was given because these ESBLs more efficiently hydrolyze cefotaxime compared with ceftazidime, and the homology between this enzyme and the TEM and SHV enzymes is only 40%. At present, CTX-M- type ESBLs have become the most popular worldwide, and more than 90 CTX-M-type enzymes have been discovered [16]. In the present study, 34 strains of ESBL-producing *Shigella* were identified as CTX-M-type ESBLs with two distinct subtypes.

Through the analysis of drug susceptibility of these two groups of ESBL-producing *S. flexneri*, we found that there was a conspicuous difference in resistance to cefepime, ceftazidime and aztreonam. The CTX-M-14 type strains were more sensitive, while the CTX-M-15 type strains appeared to have a different degree of resistance. Relative hydrolysis rate determination also showed that the CTX-M-14-type enzyme cannot hydrolyze these three drugs. This inability to hydrolyze may be related to the existence of only Ser in the 237 position [17] with a lack of Lys or Arg active groups in the 240 position.

The drug susceptibility results showed that 34 strains of ESBL-producing *S. flexneri* exhibited serious cross resistance and multidrug resistance phenomena—35.3% resistant to ciprofloxacin, 73.5% resistant to ceftazidime and cefepime, while 100% sensitive to cefoxitin, imipenem and gentamicin (patients with diarrhea are often given oral treatment in China). These results mean that there are a very limited number of antibiotics that one can choose to treat bacterial dysentery caused by ESBL-producing *Shigella*.

ESBLs are widely distributed in several bacteria. The Enterobacteriaceae bacteria is particularly beneficial for the production of resistance to antibiotics in *Shigella* because of its plasmid-mediated and integron-captured properties [18–20], which make it difficult to implement interventions that can successfully control and treat the disease. Therefore, to strengthen the monitoring and molecular epidemiological study of resistance phenotype and genotype of ESBLs-producing strains, find out the epidemic characteristics of ESBL-producing *Shigella* in the region, and provide data support for clinicians, which is of great significance for delaying the production of bacterial resistance and controlling the spread and prevalence of resistant strains in a timely and effectively.

## Abbreviations

ESBL: extended-spectrum β-lactamase
*S. flexneri*: *Shigella flexneri*
